# Hetero agressive behavior in bipolar disorder : about 30 cases

**DOI:** 10.1192/j.eurpsy.2023.1459

**Published:** 2023-07-19

**Authors:** F. Znaidi, S. Ellini, F. Fekih Romdhane, M. Cheour, R. Damak

**Affiliations:** Department of psychiatry, Razi Hospital of Manouba, Manouba, Tunisia

## Abstract

**Introduction:**

The social representations linked to bipolar disorder remain until today marked by unpredictability, danger and aggressiveness.

**Objectives:**

The objectives of this study were to identify clinical and socio-demographic characteristics of a group of patients with bipolar disorder who had committed a heteroaggressive act and to establish the relationship between these characteristics and the risk of recurrence of violence

**Methods:**

We conducted a descriptive and analytical study, including the files of 30 patients with bipolar disorder and hospitalized for heteroaggressive behavior.

We collected the data related to the socio-demographic, anamnestic and clinical characteristics of the patients.

**Results:**

The average age of patients was 40.87 years, with extremes ranging from 25 to 62 years. The patients were mostly male (60%), living in urban areas (80%) and single (60%). 66,67% of patients in our sample were professionally active.

Half of the patients in our study were consumers of alcohol or cannabis. The majority of patients were on sodium valproate (66.7%), a long-acting neuroleptic (60%) and a benzodiazepine (56.7%).

Heteroaggressive behavior was in most cases physical (63.3%), having taken place in the patient’s home (66.67%) and directed towards a family member in an impulsive context (53.33%). 63.3% of patients reoffended their act of violence.

We found statistically significant correlations between the recurrence of heteroaggressive behavior and advanced patient age (p=0.01), male sex (p=0.05), illiteracy (p=0 .04) and unemployment (p=0.04).

Our study also showed that the recidivism of violence was significantly correlated with the criminal history of the patients (p=0.04) as well as the consumption of alcohol and cannabis (p=0.01). The number of recurrences was proportionally correlated to the duration of consumption.

Recurrence of violence was also significantly higher in patients with psychiatric comorbidity (p=0.01) and poor treatment compliance (p=0.04).

Finally, the number of recurrences of heteroaggressivity was proportionally correlated to the number of hospitalizations (p= ˂10¯³).

**Conclusions:**

Heteroagressive behavior in patients with bipolar disorder is not only attributable to mental illness but also to the intertwining of several risk factors. The prevention of violence requires the identification and management of these risk factors.

**Disclosure of Interest:**

None Declared

